# What is beyond LncRNAs in breast cancer: A special focus on colon cancer-associated Transcript-1 (CCAT-1)

**DOI:** 10.1016/j.ncrna.2021.11.001

**Published:** 2021-12-01

**Authors:** Noha A. Selem, Rana A. Youness, Mohamed Z. Gad

**Affiliations:** a Biochemistry Department, Faculty of Pharmacy and Biotechnology, German University in Cairo, Egypt; b Molecular Genetics Research Team (MGRT), Pharmaceutical Biology Department, Faculty of Pharmacy and Biotechnology, German University in Cairo, Egypt; c School of Life and Medical Sciences, University of Hertfordshire Hosted By Global Academic Foundation, New Administrative Capital, 11586, Cairo, Egypt

**Keywords:** CCAT-1, lncRNAs, Breast cancer, ceRNAs, microRNAs

## Abstract

Long non-coding RNAs (LncRNAs) play a vital role in the process of malignant transformation. In breast cancer (BC), lncRNAs field is currently under intensive investigations. Yet, the role of lncRNAs as promising diagnostic and/or prognostic biomarkers and as therapeutic target/tool among BC patients still needs a special focus from the biomedical scientists. In BC, triple negative breast cancer patients (TNBC) are the unlucky group as they are always represented with the worst prognosis and the highest mortality rates. For that reason, a special focus on TNBC and associated lncRNAs was addressed in this review. Colon cancer-associated transcript 1 (CCAT-1) is a newly discovered oncogenic lncRNA that has been emerged as a vital biomarker for diagnosis, prognosis and therapeutic interventions in multiple malignancies and showed differential expression among TNBC patients. In this review, the authors shed the light onto the general role of lncRNAs in BC and the specific functional activities, molecular mechanisms, competing endogenous ncRNA role of CCAT-1 in TNBC.

## List of abbreviations

Bax: Bcl-2 Associated X-Protein
BC: Breast Cancer
BL1: Basal-Like 1
BL2: Basal-Like 2
Bmi-1: B-Cell Specific Moloney Leukemia Virus Insertion Site 1
CAEs: Cancer-Associated Endothelial Cells
CAFs: Cancer-Associated Fibroblasts
CARL-5: Cancer- Associated Region Long Non-Coding RNA-5
CCAT-1: Colon Cancer-Associated Transcript 1
ceRNA: Competing Endogenous RNA
ChIRP-Seq: Chromatin Isolation By RNA Purification
circRNAs: Circular RNAs
CLIP: Crosslinking Immunoprecipitation
CRC: Colorectal Cancer
CSCs: Cancer Stem Cells
CTCF: CCCTC-Binding Factor
EMT: Epithelial -To- Mesenchymal Transition
ENCODE: Encyclopedia Of DNA Elements
ER: Estrogen Receptor
ERK/MAPK: Extracellular Signal-Regulated Kinases/Mitogen-Activated Protein Kinases
eRNAs: Enhancer RNAs
GC: Gastric Cancer
HCC: Hepatocellular Carcinoma
HER2: Human Epidermal Growth Factor Receptor 2
HMGA2: High Mobility Group At-Hook 2
hnRNPA1: Heterogeneous Nuclear RNP A1
IARC: International Agency for Research on Cancer
IM: Immunomodulatory
Ki-67: Proliferation Index
LAR: Luminal Androgen Receptor
LC: Lung Cancer
let-7: Lethal 7
lin-4: Lineage-4
lincRNAs: Long Intergenic ncRNAs
lncRNAs: Long Non-Coding RNAs
M: Mesenchymal
MEK/ERK1/2: Mitogen-Activated Protein Kinase/Extracellular Signal-Regulated Kinase 1/2
miRNAs: MicroRNAs
MREs: MicroRNA Response Elements
mRNA: Messenger RNA
MSL: Mesenchymal Stem Like
NATS: Natural Antisense Transcript
ncRNAs: Non-Coding RNAs
nts: Nucleotides
PAM50: Prediction Analysis of Microarray
PD-L1: Programmed Death-Ligand 1
piRNAs: Piwi-Interacting RNAs
PR: Progesterone Receptor
PRC2: Polycomb Repressive Complex 2
RNase P: Ribonuclease P
RPISeq: RNA-Protein Interaction Prediction
rRNA: Ribosomal RNA
siRNAs: Small Interfering RNAs
sncRNAs: Small Or Short Non-Coding RNAs
snoRNAs: Small Nucleolar RNAs
snoRNP: Small Nucleolar Protein
SNPs: Single-Nucleotide Polymorphisms
snRNAs: Small Nuclear RNAs
SPRY4: Sprouty RTK Signaling Antagonist 4
SUV39H1: Suppressor Of Variegation 3–9 Homolog 1
TILs: Tumor-Infiltrating Lymphocytes
TMB: Tumor Mutational Burden
TME: Tumor Microenvironment
TNBC: Triple Negative Breast Cancer
TNM: Tumor Node Metastasis
tRNA: Transfer RNA
TUSC3: Tumor Suppressor Candidate 3
Wnt: Wingless/Integrated
ZFX: Zinc Finger Protein

## 1 Introduction

### 1.1 Non-coding RNAs (ncRNAs)

Over the past decades, it has been known that less than 2% of the human genome encodes for proteins through messenger RNA (mRNA), and the remaining 98% were considered as “junk” DNA [[1], [2], [3]]. However, recent studies have transformed the conception of “junk” DNA into DNA that encodes functional regulatory molecules known as non-coding RNAs (ncRNAs) [1,4]. Furthermore, it was discovered that our genome is pervasively transcribed into many ncRNAs with different lengths and structures [5]. In 1993, the discovery of the first microRNA (miRNA), lineage-4 (lin-4) was documented. Seven years later with the beginning of the 21^st^ century, the discovery of lethal 7 (let-7) miRNA in the nematode *Caenorhabditis elegans* was published [[6], [7], [8]]. Since then, numerous studies were conducted unraveling the world of ncRNAs and their respective physiological and pathological functions. So far, according to the States United States' National Institutes of Health's National Library of Medicine (PubMed), 224,290-research studies were published using the term “Non-coding RNAs” in the search bar.

NcRNAs have proved their indisputable role in several cellular processes such as chromatin remodeling, gene silencing, signal transduction, protein synthesis, transcription and post-transcriptional modifications [1,9]. Through the complex networking with DNA molecules, some proteins and even other ncRNAs or mRNAs, ncRNAs have proved to play a maestro role in curbing several signaling pathways simultaneously [9,10]. Aberrant expression of ncRNAs in several malignancies has been noticed denoting them as oncogenic drivers or tumor suppressors in several malignant contexts [11,12]. Nonetheless, it has been reported that ncRNAs could potentially act as biomarkers for diagnosis, progression, and as therapeutic targets in numerous cancers [13].

#### 1.1.1 Classification of Non-coding RNAs

**Fig. 1:**
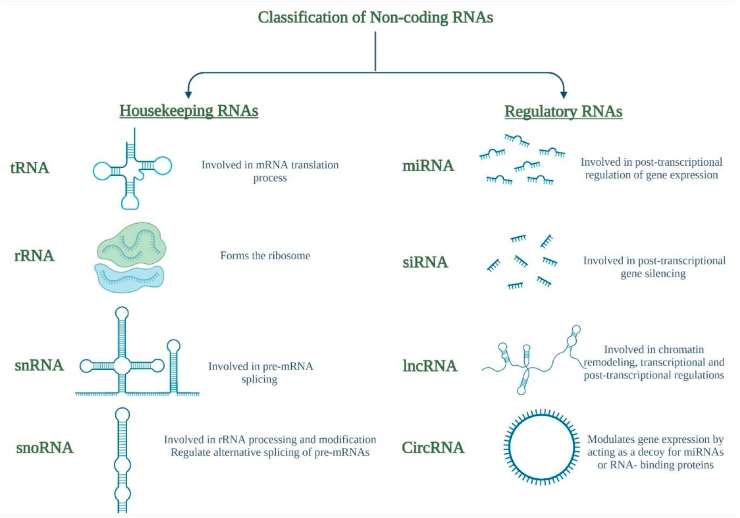
Classification of non-coding RNAs. The figure shows a schematic diagram of non-coding RNAs classes which include housekeeping and regulatory RNAs with their respective functional roles. Housekeeping RNAs include tRNA, rRNA, snRNA and snoRNA while regulatory RNAs include miRNA, siRNA, lncRNA and circRNA.

NcRNAs are mainly classified into two main categories; housekeeping and regulatory ncRNAs as represented in Fig. 1 [10]. Housekeeping ncRNAs are constitutively and ubiquitously expressed in all cell types, and are vital for several cellular functions [14]. Ribosomal RNA (rRNA), transfer RNA (tRNA), small nuclear RNAs (snRNAs) and small nucleolar RNAs (snoRNAs) are amongst those housekeeping ncRNAs [13,15]. Recently, regulatory ncRNAs have evoked researchers' interest, because of their vital regulatory functions on coding genes' expression at the epigenetic, transcriptional and post-transcriptional levels [16,17]. Regulatory ncRNAs are divided into two main groups, linear RNAs and circular RNAs (circRNAs) [13,18]. Linear regulatory ncRNAs can be further subdivided to two main categories according to their length: small or short non-coding RNAs (sncRNAs) (<200 nucleotides (nts)) and long non-coding RNAs (lncRNAs) (≥200 nucleotides) which will be the main focus of this review [10,19]. sncRNAs comprise three main classes, microRNAs (miRNAs), small interfering RNAs (siRNAs) and piwi-interacting RNAs (piRNAs) which are the most extensively studied classes [10,17,18,[20], [21], [22], [23], [24], [25], [26], [27], [28], [29]]. However, some ncRNAs exist with variable lengths such as enhancer RNAs (eRNAs) and circRNAs which could belong to both sncRNAs and lncRNAs as presented in Fig. 1 [10].

### 1.2 What are lncRNAs?

As previously described, lncRNAs are non-coding transcripts of more than 200 nts in length lacking protein-coding capacity [10,30]. According to the most recent GENCODE project gene annotation, a part of the Encyclopedia of DNA Elements (ENCODE) project consortium, almost 18,000 genes and more than 48,000 transcripts of lncRNAs in human genome are found until now [31,32]. While lncRNAs were considered for decades as “transcriptional noise” of the mammalian genome with no biological functions, currently accumulated evidence emphasized that lncRNAs are implicated in numerous biological processes [33,34]. Recent studies revealed that complex and multitude regulatory signaling pathways could be orchestrated by a single lncRNA and its prey miRNAs forming a new concept of competing endogenous RNAs (ceRNAs) [5,35]. Functionally, lncRNAs regulate transcriptional and post-transcriptional gene expression, chromatin remodeling, and cell differentiation and function [[36], [37], [38]].Moreover, deregulation of lncRNAs was found to contribute to the development of numerous diseases such as atherosclerosis, immune responses, neuronal disorders, idiopathic pulmonary fibrosis and most importantly cancer [36,39,40].

#### 1.2.1 Biogenesis of lncRNAs

LncRNAs biogenesis is comparable to the synthesis of protein coding transcripts. It takes place in the nucleus and controlled by specific stimuli that depends on different cellular contexts [12,41]. Frequently, lncRNA promoters are regulated by transcription factors and repressor proteins that activate or hinder its expression level respectively as any coding gene, also it is genomic location is often prone to several episodes of histone modifications [42]. Like mRNAs, many lncRNAs are transcribed by RNA polymerase II, the vast majority of them are spliced, 5′- capped and polyadenylated [43,44]. Minor group of lncRNAs are transcribed by RNA polymerase III [44]. Concerning lncRNAs localization, in contrast to mRNAs that are directly attached to ribosomes in the cytoplasm, lncRNAs localization is substantially different, as certain lncRNAs can occupy the chromatin, subnuclear domains, the nucleoplasm or the cytoplasm [45]. It is also worth mentioning that lncRNAs have generally lower expression levels than mRNAs and it was also distinguished that lncRNAs expression is more bound to cell type [45,46]. Finally, whereas mRNAs are primarily degraded in the cytoplasm by decapping and 5′-to-3′ exonuclease digestion, numerous unstable lncRNA transcripts are liable to the nuclear exosome or to cytosolic nonsense-mediated decay (NMD) [47].

#### 1.2.2 Classification of lncRNAs

**Fig. 2:**
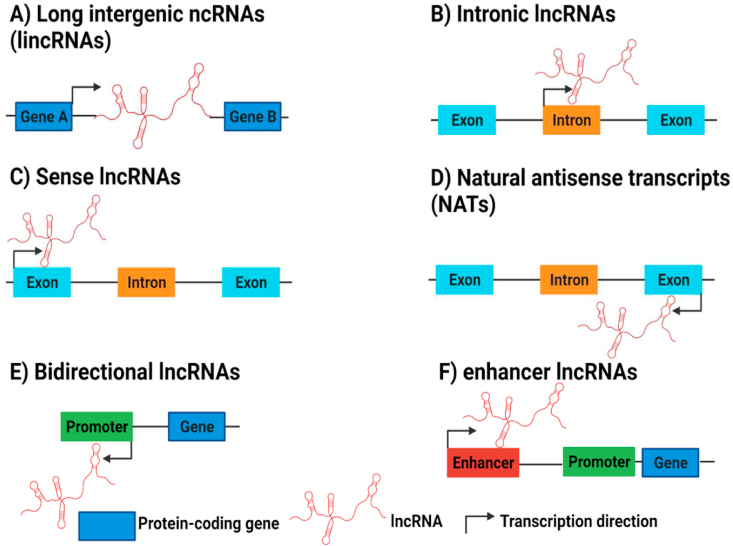
Types of LncRNAs. Based on their genomic localization, lncRNAs are classified into A) long intergenic ncRNAs (lincRNAs), B) intronic lncRNAs, C) sense lncRNAs, D) Natural antisense transcripts (NATs), E) Bidirectional lncRNAs and F) enhancer lncRNAs.

LncRNAs can be divided into several distinct classes according to their genomic localization with respect to the adjacent protein-coding genes; namely: intergenic, intronic, sense, antisense, bidirectional and enhancer lncRNAs (Fig. 2) [48,49]. Long intergenic ncRNAs (lincRNAs) exist between two protein-coding genes and transcribed independently in intergenic regions [50,51]. Intronic lncRNAs are transcribed from the introns of protein-coding genes [47]. Sense lncRNAs are transcribed from the DNA sense strand and overlap with one or more exons of protein-coding genes [52]. Natural antisense transcript (NATs), which exhibit a large proportion of lncRNAs, are transcribed from the DNA antisense strand and may complement partially or completely the transcripts on the opposite strand [53]. Bidirectional lncRNAs are transcribed from the same promoter region with protein-coding genes, nonetheless in the opposite direction [53,54]. Lastly, enhancer lncRNAs are transcribed from promoter enhancer regions of protein - coding genes [34,47,53]. It was illuminated that distinct mechanisms are encompassed in the biogenesis of lncRNAs such as ribonuclease P (RNase P) cleavage to create mature ends, the formation of snoRNA and small nucleolar protein (snoRNP) complex caps at their ends and circular structure production [55]. Generally, lncRNAs biogenesis and regulation are still not fully identified, yet in the coming years with the advancement of numerous techniques, for example ChIRP-Seq (Chromatin Isolation by RNA purification), crosslinking immunoprecipitation (CLIP), RNA structure mapping, ribosome profiling, targeted genome engineering by CRISPR and advanced genetic screens, information regarding lncRNAs biogenesis and functions will be much more deepened [56,57].

#### 1.2.3 Mechanism of action of lncRNAs

Until 2010, most of the discovered lncRNAs were of unknown and non-tangible mechanism of action. Until recently, the number of lncRNAs with known mechanism of action has been steadily increasing in the past few years [43]. They can serve as scaffolds, decoys or signals [54]. It was further revealed that lncRNAs can be sub-classified into *cis*-lncRNAs that regulate the expression of close proximity genes, and *trans*-lncRNAs that control distant genes expression [36,58]. Later, it was unveiled that lncRNAs could act as a catalyst in several epigenetic modifications mainly by recruiting chromatic remodeling complex to a specific chromatin location [59] where it was revealed that almost 38% of the discovered lncRNAs are found bound to the polycomb repressive complex 2 or the chromatin modifying proteins, CoREST and SMCX while others were found to be bound to trithorax chromatin-activating complexes and/or activated chromatin [60]. For instance, this could be fully descriptive in case of XIST, HOTAIR and ANRIL lncRNAs where XIST was found to have a leading role in the X chromosome inactivation through the ablation of promotor region of XIST [61]. For HOTAIR, it was reported to act as scaffold coordinating the direction and targeting of specific loci for reprogramming the chromatin state while for ANRIL it was found to bind to polycomb-group proteins namely PRC1 and PRC2 [59]. Nonetheless, more recent studies have showed that lncRNAs could act as modifiers affecting transcription factors' respective activities. For instance, Evf2 lncRNA was reported to be transcribed from a conserved distal enhancer and recruits DIX2 transcription factor to the same enhancer region to induce the expression of adjacent protein coding genes [62]. Other recent lncRNAs such as sONE lncRNA discovered by our research group had actually the potential to directly affect the transcription factors TP53 and cMYC and their respective target genes [62]. Furthermore, the ability of lncRNAs to identify complementary sequences on other coding genes (mRNAs) or non-coding genes (other ncRNAs) make it capable to mediate its actions via post-transcriptional routes such as capping, splicing, editing, transport, translation, degradation, and stability at various control sites or even sponging effects to other ncRNAs such as miRNAs [21,24,35,59]. For instance, MALAT-1 could dually affect coding and non-coding genes as it mainly affects the splicing process for mRNAs through interacting with several splicing factors and at the same time it was reported to have extensive sponging capabilities on several tumor suppressor miRNAs such as miR-17-5p [63] and miR-486-5p [64].

#### 1.2.4 LncRNAs in oncology

Recently, lncRNAs have emerged as vital players in the field of cancer biology through their ability to regulate every single hallmark of cancer, such as cellular proliferation, apoptosis, migration, invasion, immune escape, epithelial to mesenchymal transition and even metastasis [65]. Additionally, it was showed that lncRNAs’ expression depends on the cancer origin, giving them a strong rationale to be implicated as possible biomarkers for diagnosis, prognosis and/or therapeutic targets of several cancer contexts [66]. It is also worth mentioning that such aberrant expression of lncRNAs plays a prominent role in the tumorigenic process of several solid malignancies including breast cancer (BC).

### 1.3 Breast cancer (BC)

BC is the most common malignancy worldwide [67]. According to the latest Globocan statistics that conducted by the International Agency for Research on Cancer (IARC), an estimated 2.3 million new cases were diagnosed with BC in 2020 [67]. Furthermore, BC is considered the fifth-leading cause of cancer-related mortality with almost 685,000 annual deaths worldwide. An enormous attention was conferred toward BC subtypes, since they were associated with variations in prognosis and treatment responses [68,69]. With the advent of molecular analysis methods, Perou and Sorlie had presumed the intrinsic molecular classification of BC based on Prediction Analysis of Microarray (PAM50) using 50-gene expression profiling in 2000 [[70], [71], [72]]. Their study categorized BC into 4 different molecular subtypes, which are luminal A, luminal B, human epidermal growth factor receptor 2 (HER2) enriched and basal-like triple negative breast cancer (TNBC) (Table 1**)** [69,73]. TNBC can be subdivided into 6 distinct molecular subtypes including, basal-like 1 (BL1), basal-like 2 (BL2), immunomodulatory (IM), mesenchymal (M), mesenchymal stem like (MSL) and luminal androgen receptor (LAR) [74]. Despite the profound advances in the early detection and precise management of BC over the past decades, BC is still liable to very high risks of recurrence, metastasis and treatment resistance [75,76].

**Table 1:** **BC molecular subtypes** [[77], [78], [79], [80], [81]].

Molecular subtype	Gene profiling	Frequency and prognosis	Treatment
Luminal A	ER^+^ and/or PR^+^, HER2^−^and low ki-67 < 14%	50% of invasive BC Good prognosis	Endocrine therapy used alone frequently Chemotherapy
Luminal B	ER^+^ and/or PR^+^, HER2^+/−^and high ki-67 ≥ 14%	20% of invasive BC Poorer prognosis in contrast to luminal A	Endocrine therapy Chemotherapy Anti-HER2 antibody “in case of HER2^+^”
HER2-enriched BC	ER^−^, PR^−^, HER2^+^ and high ki-67 ≥ 14%	15% of invasive BC Poor prognosis	Anti-HER2 antibody Chemotherapy
Triple negative breast cancer (TNBC)	ER^−^, PR^−^, HER2^-^ and high ki-67 ≥ 14%	15% of invasive BC Worst prognosis	Chemotherapy only

ER; estrogen receptor, PR; progesterone receptor, HER2; human epidermal growth factor 2, Ki-67; proliferation index.

#### 1.3.1 LncRNAs as functional players in BC

According to the States United States' National Institutes of Health's National Library of Medicine (PubMed), upon using “Breast Cancer” and “lncRNAs” as the descriptors in the search bar 2227 publications are obtained. Table 2 summarizes the available information regarding functional lncRNAs modulating BC hallmarks. The inclusion criteria of literature include published data that are complete, relevant, available online, in English, and with detailed information about participants, methods, and analyses. Data collection was done during June 2021, and data abstracted was in the form of descriptive information, covering the type of validation studies used, techniques, and findings or reported effects. Bias was limited through the evaluation of the studies through their internal validity rather than the authors' claimed conclusions. It was quite evident that lncRNAs are promising candidates as novel cancer biomarkers for diagnosis, prognosis and targeted therapy for BC patients [36,[82], [83], [84], [85]].

**Table 2:** Functional long non-coding RNAs modulating breast cancer hallmarks.

LncRNA	Genomic location	Functional Role	Affected BC Hallmarks	Validation Study	References
HOTAIR (Hox antisense intergenic RNA)	12q13.13	Oncogene	Proliferation	*In vivo* *In vitro*	[[86], [87], [88], [89], [90]]
			Migration		
			Invasion		
			Metastasis		
			EMT		
CCAT-1 (Colon Cancer-Associated Transcript 1)	8q.24.21	Oncogene	Proliferation	*In vivo* *In vitro*	[[91], [92], [93]]
			Migration		
			Invasion		
			Metastasis		
H19	11p15.5	Oncogene	Invasion	*In vivo* *In vitro*	[24,[94], [95], [96]]
			Metastasis		
			EMT		
			Migration		
			Proliferation		
MVIH (LncRNA associated with microvascular invasion in hepatocellular carcinoma)	10q22-q23	Oncogene	Proliferation	*In vivo* *In vitro*	[97,98]
			Invasion		
			Apoptosis		
SPRY4-IT1 (SPRY4 intronic transcript 1)	5q31.3	Oncogene	Proliferation	*In vivo* *In vitro*	[99,100]
			Apoptosis		
PCAT-1 (Prostate cancer associated transcript-1)	8q24.21	Oncogene	Proliferation	*In vitro*	[101,102]
HULC (Highly upregulated in liver cancer)	6p24.3	Oncogene	Metastasis	*In vivo* *In vitro*	[103,104]
			Migration		
			Invasion		
			Apoptosis		
UCA1 (urothelial carcinoma-associated 1)	19p13.12	Oncogene	Apoptosis	*In vivo* *In vitro*	[[105], [106], [107], [108]]
			Proliferation		
HEIH (Hepatocellular Carcinoma Up-Regulated EZH2-Associated Long Non-Coding RNA)	5q35.3	Oncogene	Proliferation	*In vivo* *In vitro*	[41,109]
			Migration		
			Metastasis		
ANRIL (antisense non-coding RNA in the INK4 locus)	9p21.3	Oncogene	Proliferation	*In vivo* *In vitro*	[110]
			Apoptosis		
LncRNA-ATB (Long noncoding RNA activated by transforming growth factor-beta)	14:19,858,667–19,941,024	Oncogene	EMT	*In vivo* *In vitro*	[[111], [112], [113], [114]]
			Invasion		
			Metastasis		
ZEB2-AS1 (zinc finger E-box binding homeobox 2 antisense RNA 1)	2q22.3	Oncogene	Proliferation	*In vivo* *In vitro*	[115]
			Metastasis		
			EMT		
BCAR4 (Breast cancer anti-estrogen resistance 4)	16p13.13	Oncogene	Metastasis	*In vivo* *In vitro*	[116]
			Proliferation		
LincRNA-ROR (Long Intergenic Non-Protein Coding RNA, Regulator Of Reprogramming)	18q21.31	Oncogene	Proliferation	*In vivo* *In vitro*	[[117], [118], [119]]
			Invasion		
			EMT		
			Metastasis		
NEAT1 (Nuclear Enriched Abundant Transcript 1)	11q13.1	Oncogene	Migration	*In vivo* *In vitro*	[[120], [121], [122], [123]]
			Invasion		
			Proliferation		
			EMT		
Linc00152	2p11.2	Oncogene	Invasion	*In vivo* *In vitro*	[124]
			Metastasis		
			Apoptosis		
DSCAM-AS1 (Down Syndrome Cell Adhesion Molecule antisense 1)	21q22.2	Oncogene	Proliferation	*In vivo* *In vitro*	[125,126]
			EMT		
			Apoptosis		
MALAT-1 (Metastasis Associated Lung Adenocarcinoma Transcript 1)	11q13.1	Controversial	Proliferation	*In vivo* *In vitro*	[35,[127], [128], [129], [130]]
			Migration		
			Metastasis		
			Angiogenesis		
PANDAR (promoter of CDKN1A antisense DNA damage-activated RNA)	6p21.2	Controversial	Apoptosis	*In vivo* *In vitro*	[131,132]
			Proliferation		
			Invasion		
			EMT		
XIST (X inactive specific transcript)	Xq13.2	Controversial	Migration	*In vivo* *In vitro*	[133,134]
			Invasion		
PLNCRNA-1 (Prostate cancer-up-regulated long noncoding RNA 1)	21q22.12	Tumor suppressor	Proliferation	*In vivo* *In vitro*	[135]
			Apoptosis		
GAS5 (Growth arrest-specific transcript 5)	1q25.1	Tumor suppressor	Metastasis	*In vivo* *In vitro*	[136,137]
			Apoptosis		
			Proliferation		
			Invasion		
NBAT1 (Neuroblastoma Associated Transcript 1)	6p22.3	Tumor suppressor	Metastasis	*In vivo* *In vitro*	[138]
			Invasion		
			Migration		
MEG3 (Maternally expressed gene 3)	14q32.2	Tumor suppressor	Proliferation	*In vivo* *In vitro*	[139,140]
			Apoptosis		
sONE	7q36	Tumor suppressor	Proliferation	*In vitro*	[22]
			Migration		
			Invasion		
LincRNA-p21 (Long intergenic noncoding RNA p21)	6p21.2	Tumor suppressor	Apoptosis	*In vivo* *In vitro*	[141,142]
			Migration		
			Invasion		
			Proliferation		
PTENP1 (PTEN pseudogene-1)	9p13.3	Tumor suppressor	Proliferation	*In vivo* *In vitro*	[[143], [144], [145]]
			Invasion		
			Migration		
			Apoptosis		

#### 1.3.2 A special focus on TNBC

##### 1.3.2.1 TNBC: An orphan subtype with respect to therapeutic algorithms

TNBC is characterized by lacking hormone receptors (ER and PR) expression, absence of HER2 and high expression levels of Ki-67 protein (≥14%). TNBC accounts for 15–20% of all invasive BC cases and considered the most aggressive and heterogeneous subtype with higher tendency of recurrence and distant metastasis resulting in high mortality [69]. TNBC has a weak response to hormone therapy and HER2 antibodies [146]. The clinical management of TNBC is a major remedial challenge due to its heterogeneity and paucity of precise targeted therapy. Therefore, a better understanding of the molecular engines fueling TNBC development and progression is an urgent biomedical need. Recent studies have demonstrated that aberrant expression of lncRNAs significantly plays important roles in TNBC cell proliferation, angiogenesis, cell cycle, migration, invasion, apoptosis, drug resistance and tumorigenicity [147]. Nonetheless, lncRNAs were also found to act as stable promising non-invasive diagnostic and prognostic markers for TNBC patients. For instance, our research group was the first to shed the light onto sONE lncRNA as a restrictedly expressed lncRNA in TNBC patients [22]. Moreover, it showed a differential expression pattern between old and young TNBC patients and significant correlation with different disease parameters [22].

##### 1.3.2.2 TNBC: A hot tumor with respect to its immune-condensed tumor microenvironment

**Fig. 3:**
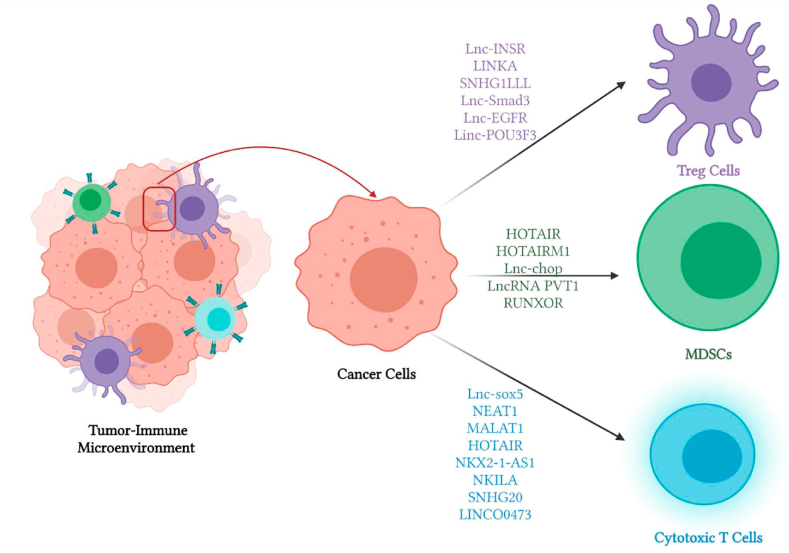
LncRNAs are fundamental players at the tumor-immune microenvironment. This figure represents a snap-shot for the crosstalk between cancer cells and immune cells at the tumor immune microenvironment. The figure comprises lncRNA that modulate the infiltrated immune cells at the TIME such as T regulatory cells, myeloid derived stem cells and cytotoxic T cells.

Unveiling another side of TNBC revealed that those types of tumors are characterized by distinctive tumor immune microenvironment (TIME) that has an indisputable role in inducing cellular proliferation, angiogenesis, drug resistance, immune evasion and inhibition of apoptosis of the TNBC cells. TIME of TNBC comprises several stromal cells such as cancer-associated endothelial cells (CAEs), cancer stem cells (CSCs), cancer-associated fibroblasts (CAFs) and infiltrated immune cells, all of which are comprised in an intricate network with tumor cells to directly fuel/trim tumor progression [148]. Collectively, this underscores that potential of TIME members to act as potential therapeutic targets for TNBC patients [149]. Most of lncRNAs localize in the nucleus, however a large fraction is present as circulating lncRNAs after their transmission via exosomes and thus modulating the TIME of TNBC [150]. Emerging evidence proved that lncRNAs can stimulate TIME and contribute in tumor-stroma crosstalk (Fig. 3) [148].

##### 1.3.2.3 Roles of lncRNAs in TNBC

**Fig. 4:**
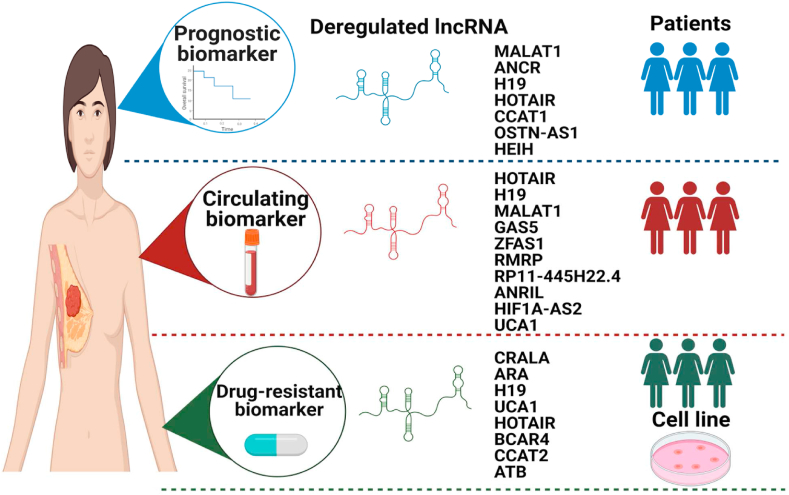
Potential clinical application of deregulated lncRNAs in TNBC. LncRNAs with aberrant expression can act as potential prognostic, circulating and drug-resistance biomarkers for TNBC patients.

Numerous lncRNAs are involved in mammary gland development and accordingly BC evolution [151]. LncRNAs can regulate TNBC through multidisciplinary molecular roles, such as cellular proliferation, invasion, metastasis, apoptosis and drug resistance [152,153]. Yet, the definite mechanisms of how lncRNAs can contribute to these aspects remain largely enigmatic [154,155]. LncRNAs may act as potential diagnostic, prognostic biomarkers and therapeutic targets in BC (Fig. 4) [155,156]. In addition, lncRNAs differential expression was postulated to discriminate between TNBC and non-TNBC subtypes [157]. LncRNAs can be categorized into oncogenes or tumor suppressor lncRNAs according to their functions and expression patterns in BC patients [15,158,159]. Colon Cancer- Associated Transcript 1 (CCAT-1) is one of the aberrantly expressed lncRNAs among TNBC patients [91]. Nonetheless, our research group has recently found its induced expression among TNBC patients and cell lines versus non-TNBC patients and hormonal receptor positive BC cell lines [160].

### 1.4 Colon Cancer- Associated Transcript 1 (CCAT-1) LncRNA

#### 1.4.1 CCAT-1 structure and chromosomal location

**Fig. 5:**
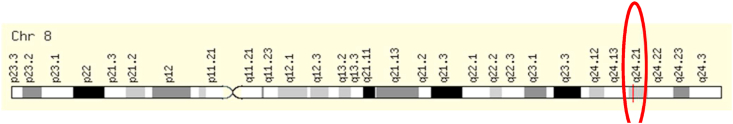
Chromosomal location of CCAT1 gene (8q24.21). Schematic representation of CCAT-1 gene locus; chromosomal mapping showed that CCAT-1 gene is located on somatic chromosomal 8, long arm, region 24, band 21 (8q24.21).

LncRNA CCAT-1 is encoded by CCAT-1 gene. It is also known as cancer-associated region long non-coding RNA-5 (CARL-5). CCAT-1 is a recently identified lncRNA with a length of 2628 nucleotides and mapped to chromosome 8q24.21 (Fig. 5). It is worth mentioning that CCAT-1 lncRNA is in a proximity to the proto-oncogenic transcription factor c-Myc [161,162]. This chromosomal location was distinguished as a ‘hot spot’ harboring single-nucleotide polymorphisms (SNPs) that cause numerous genetic alternations, and consequently aid in the development of several malignances including colon, prostate, and breast cancers [161,163,164]. CCAT-1 is upregulated in nearly all stages of colorectal cancer (CRC) and its overexpression usually curtails poor prognosis and low survival rate among CRC patients [165]. It is an enhancer-derived RNA that is transcribed from a distal enhancer, 515 kb upstream of the c-Myc gene. It is also worth noting that CCAT-1 consists of two exons and a poly-A tail and is mainly expressed in the nucleus [166]. It was elucidated that CCAT-1 in colon cancer cells is localized at its site of transcription which is vital for mediating the long-range chromatin interactions between CCAT-1 gene and c-Myc in conjunction with an enhancer 335 kb upstream of c-Myc. In this way, CCAT-1 transcriptionally activates c-Myc in a *cis*-acting manner; therefore, depletion of CCAT-1 could reduce the transcription of c-Myc gene [161,167]. CCAT-1 gene can be transcribed into two isoforms: the long isoform CCAT-1-L and the short isoform CCAT-1-S [168]. While CCAT-1-L is highly expressed in the nucleus, CCAT-1-S isoform is solely expressed in the cytoplasm of various tumors [169,170]. It was proposed that CCAT-1-S may be developed from CCAT-1-L and a positive correlation may exist, as the downregulation of CCAT-1-L resulted in a concurrent disruption of CCAT-1-S levels [170].

#### 1.4.2 CCAT-1 as an oncogenic driver in several malignant contexts

CCAT-1 lncRNA is significantly upregulated and act as an oncogenic lncRNA in several types of cancers such as CRC, gastric cancer (GC), hepatocellular carcinoma (HCC), lung cancer (LC) and BC [166,169,171,172]. CCAT-1 was found to be implicated in various biological processes, including proliferation, migration, invasion, cell cycle, apoptosis, epithelial -to- mesenchymal transition (EMT), survival and chemo-resistance of cancer cells [168,173]. Additionally, the aberrant expression of CCAT-1 was positively correlated with tumorigenesis, tumor size, tumor node metastasis (TNM) stage, differentiation, angiogenesis, lymph node metastasis, overall survival, recurrence-free survival, and treatment outcome [168,174]. Consequently, CCAT-1 has emerged as a vital biomarker for diagnosis, prognosis, and therapeutic interventions in multiple malignancies [91,173,174].

### 1.5 CCAT-1 in BC

**Fig. 6:**
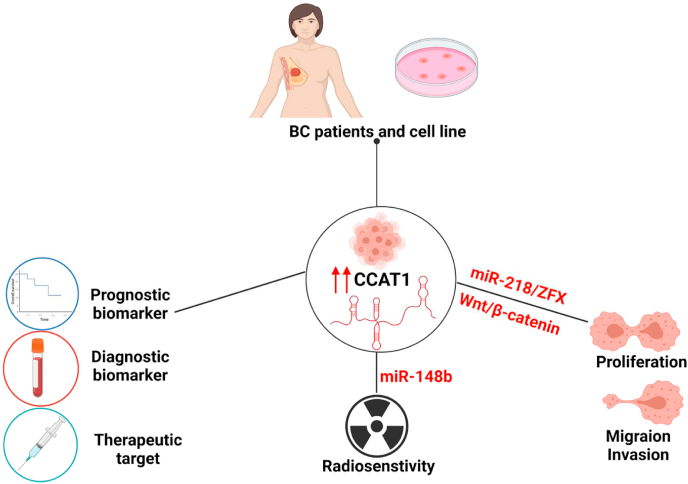
Potential of CCAT1 as diagnostic, prognostic biomarker, and therapeutic target in Breast Cancer. CCAT1 is highly expressed in BC. CCAT1 acts as a potential prognostic, diagnostic biomarker, and therapeutic target for BC. CCAT1 can regulate miR-148b and modify the radiosensitivity of BC. Additionally, it was revealed that CCAT1 can promote proliferation, migration, and invasion through targeting miR-218/ZFX axis in TNBC and activating Wnt/β-catenin signaling pathway in BC cells.

CCAT-1 is markedly up-regulated in BC tissues compared to its normal counterparts [175]. Such overexpression of CCAT-1 was directly associated with TNM stage and lymph node metastasis, thus referring that CCAT-1 may play crucial roles in BC tumorigenesis and metastasis [175]. Its high expression may also exerts a negative influence on the overall survival and progression-free survival; hence CCAT-1 is considered a non-invasive prognostic biomarker for BC patients [175]. Using RNA-protein interaction prediction (RPISeq) software it was asserted that CCAT-1 has direct correlation with several clinical biomarkers such as HER2, ER, PR [176]. Another study showed that silencing of CCAT-1 significantly induced miR-148b and improved the radio-sensitivity of BC cells [177]. A recent study had reported that CCAT-1 can activate Wnt/β-catenin signaling pathway and thereby promotes breast cancer stem cell function (BCSC) and BC progression [178]. Collectively, the available literature highly supports the association of CCAT-1 with BC tumorigenesis and highly proposes it as a promising diagnostic and prognostic biomarker and therapeutic target for BC patients (Fig. 6) [178].

### 1.6 CCAT-1 in TNBC

Concerning TNBC, the most aggressive BC subtype, it was shown that CCAT-1 promotes TNBC cellular proliferation, migration and invasion through targeting a novel oncogenic axis; miR-218/ZFX axis [93]. Nonetheless, it was recently reported by our research group that CCAT-1 showed a differential expression between different BC subtypes, showing the highest expression level among TNBC patients if compared to other BC subtypes [179]. Moreover, we and others have recently reported the elevated expression of CCAT-1 in TNBC cell lines (MDA-MB-231, MDA-MB-436 and MDA-MB-468) compared to hormonal receptor positive (MCF-7) cells and normal human epithelial cells (MCF-10) [93,179,180]. It is also worth noting that CCAT-1 expression level was directly correlated with lymphnode metastasis, proliferation index (Ki-67) and tumor size among TNBC patients [160,180].

#### 1.6.1 Molecular circuits regulated by CCAT-1 lncRNA: A special focus on TNBC

**Fig. 7:**
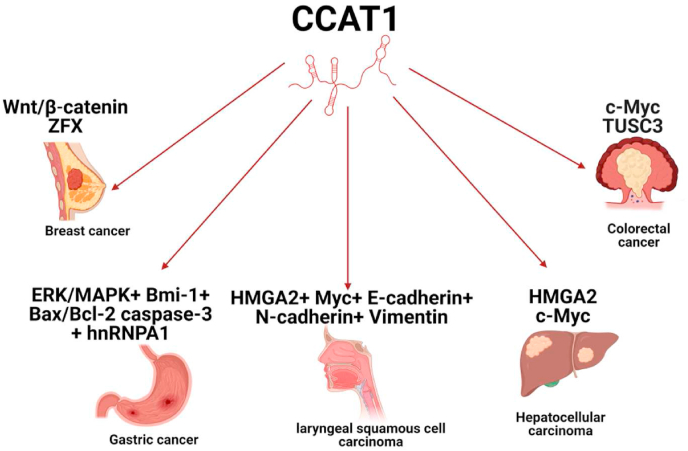
Central Role of CCAT-1 as an oncogenic driver in several solid malignancies. CCAT-1 was found to promote colorectal cancer tumorigenesis through regulating TUSC3 and c-Myc, hepatocellular carcinoma progression via HMGA2 and c-Myc, laryngeal squamous cell carcinoma by targeting HMGA2, Myc, E-cadherin, N-cadherin and Vimentin, gastric cancer development via targeting ERK/MAPK, Bmi-1, Bax/Bcl-2 caspase-3 and hnRNPA1 and BC progression via Wnt/β-catenin and ZFX signaling pathways. TUSC3, tumor suppressor candidate 3; hnRNPA1, heterogeneous nuclear RNP A1; Bmi-1, B-cell specific moloney leukemia virus insertion site 1; ERK/MAPK, extracellular signal-regulated kinases/mitogen-activated protein kinases; Bax, Bcl-2 Associated X-protein; HMGA2, High Mobility Group AT-Hook 2; ZFX, zinc finger protein; Wnt, wingless/integrated.

CCAT-1 was found to be involved in the activation of several oncogenic signaling pathways in different cellular contexts such as mitogen-activated protein kinase/extracellular signal-regulated kinase 1/2 (MEK/ERK1/2) and phosphatidylinositol 3-kinase (PI3K/AKT) as shown in Fig. 7 [181]. It is also worth noting that CCAT-1 functions as a scaffold for Polycomb repressive complex 2 (PRC2), a suppressor of variegation 3–9 homolog 1 (SUV39H1), and epigenetically modifies the histone methylation of the tumor suppressor sprouty RTK signaling antagonist 4 (SPRY4) promoter [182]. It is also worth mentioning that our research group has recently highlighted the sponging capabilities of CCAT-1 in sequestering the tumor suppressor miRNAs let-7a and miR-17-5p in TNBC cell lines as a possible proposed ceRNA mechanism by which CCAT-1 acts as an oncogenic lncRNA in TNBC [160,179].

#### 1.6.2 Double positive feedback loops between CCAT-1 and c-Myc: A special focus on TNBC

It was reported that c-Myc can directly bind to the E-box element in the promoter region of CCAT-1, and by this binding c-Myc could enhance the transcription and expression of CCAT-1 [[183], [184], [185]]. Interestingly, it was also elucidated that CCAT-1-L is located within a super-enhancer region and encompassed in the conservation of chromatin looping interactions between the c-Myc promoter and its enhancers, promoting c-Myc expression through the interaction with CCCTC-Binding factor (CTCF) [170]. Moreover, it was found that CCAT-1 can regulate c-Myc expression post-transcriptionally via acting as a sponge for tumor suppressor miRNAs that directly target c-Myc [186]. Therefore, double-positive feedback loops can be plotted between CCAT-1 and c-Myc for enhancing the expression of each other and increasing the aggressiveness of cancer cells [187]. Noteworthy, this positive feedback loop has been evidenced among TNBC patients where a direct correlation of CCAT-1 and c-Myc expression levels was reported among TNBC patients [180]. Nonetheless, such positive feedback loop was reported in MDA-MB-231 orchestrated by let-7a building up a novel player in the intricate crosstalk between CCAT-1 and c-Myc in TNBC cells [180].

#### 1.6.3 CCAT-1 in Immuno-oncology

Since TIME has been highlighted as an important factor in the TNBC tumorigenic equation, it was interesting to unravel the role of CCAT-1 in the immune-oncology sector. Studies focusing on the role of CCAT-1 in the TIME are rare. Yet, a recent study by Liu et al. reported that CCAT-1 was highly expressed in M1 macrophages when compared to M2 and M0 macrophages indicating that CCAT-1 may serve as a significant biomarker in macrophage polarization. They also reported that silencing of CCAT-1 promoted M2 macrophages polarization and prostate cancer cell invasion through induction of miR-148a expression level which consequently reduces the expression of PKCζ [188]. TNBC is well-known as a hot immunogenic tumor due to its higher level of tumor-infiltrating lymphocytes (TILs) and tumor mutational burden (TMB) compared to other BC subtypes [189]. Furthermore, it has been reported that TNBC cells highly express programmed death-ligand 1 (PD-L1), an immune checkpoint molecule that contributes to immune evasion on their surface when compared to other non-TNBC [190]. Our research group is currently focusing on the implications of CCAT-1 on the immunogenic and oncologic profiles of TNBC patients through a circuit of ncRNAs. We have proved that CCAT-1 acts as a potential regulator for PD-L1 through sponging miR-17-5p in MDA-MB-231 TNBC cell line [160,179]. Other reports showed that CCAT-1 can act as an oncogenic lncRNA in TNBC via stimulating MDA-MB-231 cellular proliferation, clonogenicity and migration capacity [191].

#### 1.6.4 A Snapshot of CCAT-1 lncRNA as a potential ceRNA molecule

CCAT-1 represents an oncogenic drive for most of cancer cells. This is achieved through modulating an array of downstream signaling pathways as previously highlighted. Recent studies had shed the light onto an intertwined crosstalk between CCAT-1 and numerous miRNAs and their preys (targets). In such intricate crosstalk, CCAT-1 acts as a ceRNA through sponging miRNAs, regulating their downstream targets in various cancers (Table 3) [168]. ceRNAs theory refers to the concept that a regulator of miRNA such as lncRNAs, circular RNA, pseudogene RNA can sponge and bind to miRNAs competitively to indirectly regulate their respective mRNA targets building a novel lncRNA-miRNA-mRNA axis [192]. Thus, the discovery of lncRNA-miRNA network not only confers the possibility of an additional level of post-transcriptional regulation, but also dictates a reconsideration of the existing regulatory pathways involved in tumor initiation and progression [193].

**Table 3:** CCAT-1 as ceRNA molecule in different malignant contexts.

Cancer type	miRNAs	Reference
Colorectal cancer	miR-181b-5p	[194]
Hepatocellular carcinoma	let-7	[186]
Gastric carcinoma	miR-490	[195]
	miR-219-1	[196]
Laryngeal squamous cell carcinoma	let-7	[187]
	miR-218	[197]
Breast cancer	miR-148b	[177]
	miR-218	[93]
	miR-17-5p	[160]
	Let-7a	[179]
Gallbladder cancer	miR-218-5p	[198]
Glioma	miR-181b	[199]
Acute myeloid leukemia	miR-155	[200]
Thyroid cancer	miR-143	[201]
Prostate cancer	miR-148a	[188]
Melanoma	miR-33a	[202]
Cholangiocarcinoma	miR-152	[203]
Osteosarcoma	miR-148a	[204]
Ovarian cancer	miR-1290	[205]
	miR-152	[206]
	miR-130b	[206]
	miR-490-3p	[207]
Multiple myeloma	miR-181a-5p	[208]
Non-small cell lung cancer	miR-130a-3p	[209]
Esophageal squamous cell carcinoma	miR-7	[182]
Endometrial carcinoma	miR-181a-5p	[210]

## 2 Conclusion

In conclusion, identifying the underlying molecular engines fueling BC progression and metastasis is a task that perplexed many researchers worldwide. In this review, we spot the light onto a key player in the game; CCAT-1 lncRNA.CCAT-1 is an upregulated lncRNA in BC and in TNBC patients in particular. This overexpression is associated with several disease parameters such as TNM stage, tumor size, ki-67 and lymph node metastasis. Yet, it was clear that correlation of CCAT-1 and BC stages, severity and resistance to therapy is still to be explored. In this manuscript, a detailed dissection of CCAT-1 lncRNA role in oncology in general and BC in particular was plotted. CCAT-1 was proved to have a key role in modulating several canonical and non-canonical oncogenic signaling pathways in BC. Yet, it is important to spot the light onto the scarcity of literature unraveling the potential role CCAT-1 in immunoncology. Therefore, CCAT-1 lncRNA is a still novel ncRNA that warrants further study with regard its network interactions with miRNAs, target genes, downstream signaling pathways and its correlation with the immunogenic profile of BC in terms of immune ligands, cytokines and interleukins secreted from BC cells.

## Declaration of competing interest

The authors declare no conflict of interest.
